# Visual-Spatial Search in Neglect Syndrome as a Function of the Number of Stimuli in the Hemifields

**DOI:** 10.3390/healthcare12232387

**Published:** 2024-11-28

**Authors:** Nataliya Varako, Maria Kovyazina, Daria Yurina, Victoria Propustina, Georgiy Stepanov, Svetlana Vasilyeva, Vadim Daminov, Anatoliy Skvortsov, Maria Baulina, Yuri Zinchenko

**Affiliations:** 1 Faculty of Psychology, Lomonosov Moscow State University, Moscow 125009, Russia; kms130766@mail.ru (M.K.); yurinadd@my.msu.ru (D.Y.); vp4399@gmail.com (V.P.); stepanov.georgiy99@gmail.com (G.S.);; 2 Research Center of Neurology, Moscow 125367, Russia; 3 Federal Scientific Center of Psychological and Multidisciplinary Research, Moscow 125009, Russia; 4 Medical Rehabilitation Clinic, Pirogov National Medical and Surgical Center, Moscow 105203, Russia

**Keywords:** neglect syndrome, stroke, subsequent search misses, top-down and bottom-up approach, visuospatial search

## Abstract

Background: Neglect syndrome is a serious condition that often affects the ability to perform visual-spatial search tasks, interfering with the ability to detect stimuli on the left side of space. A number of factors can affect the success of visual search in patients with neglect syndrome, including visual field load. The purpose of this study is to investigate how the number of stimuli in the right visual hemifield influences the efficiency of visual search in the left (neglected) hemifield, hypothesizing that an increased object load on the right side may impair search performance on the left. Methods: The sample comprised 30 patients with neglect syndrome as the target group and 20 patients with right hemisphere damage but no signs of hemispatial neglect as the control group. This study employed several neuropsychological tests, including neuropsychological examination according to the scheme of A.R. Luria. The SPSS 23.0 software was used for statistical analysis of the data. Results: The Red Shapes test revealed a significant decrease in the number of stimuli detected in both the right and left visual hemifields across successive series (*p* < 0.001) in patients within the target group. No significant differences were observed in the participants of the control group. This study’s results may be explained by the phenomenon of SSM (subsequent search misses) and the theory of attentional resource depletion during visual search tasks. These results indicate the need for further research into the features of visual search under various conditions, including the load and structuredness of the visual field. Conclusions: This study confirmed that the number of elements in the right visual hemifield influences the ability to detect elements in the left hemifield in patients with left-sided neglect, as demonstrated using the Red Shapes test.

## 1. Introduction

Unilateral spatial neglect syndrome [1], which occurs clinically in cases of localized brain injury, is one of the most disabling impairments. According to T. Nijboer and colleagues [2], this syndrome persists in approximately 40% of patients one year after the onset of symptoms. One of the main symptoms experienced by these patients is impaired visual search.

Visual object search is a visual search process that focuses on identifying a previously requested target among distractors that differ from the target by a unique visual feature such as color, shape, orientation, and size [3,4]. Visual search is a type of perceptual task that requires a lot of attention and typically involves actively scanning the visual environment in search of a particular object or objects among distractors [3].

Visual search tasks have been used to investigate attention in the visual domain [5]. Visual search tasks are often associated with the biased competition model of visual attention [6], according to which attention is limited, and target stimuli compete with distractors for the available capacity during visual search. There are two lines of information processing: bottom-up and top-down. In the first case, more attention is paid to simple stimulus features that distinguish stimuli from the background. In the second case, attention is directed toward more complex features that make it possible to relate stimuli to the task at hand and assess the relevance of current behavior; the choice is initially determined by the observer’s goals; the shift of attention occurs only when it corresponds to a specific goal [7,8]. In this context, one of the questions is the possibility and conditions under which top-down regulation of search would make this search more efficient [9]. Search is considered efficient when its speed does not depend on the number of stimuli in the visual field and inefficient when search speed decreases as the number of stimuli increases [10]. Search is also influenced by the location of objects, their properties, and the subject’s own characteristics, such as motivation [7].

Among all types of visual search tasks, those involving two or more target stimuli occupy a special place, as they are closest to real-life situations. When performing such tasks, subjects experience the phenomenon of “subsequent search misses” (SSM) [11]. Initially, this phenomenon was called “satisfaction of search”, in which the subject successfully completes the search of the first target stimulus but misses the second, feeling satisfied with the result achieved [12].

H.-O. Karnath [13] suggests that neglect syndrome is associated with a dissociation between bottom-up and top-down attentional networks. Patients with neglect are more likely to have attentional deficits associated with low-level, involuntary, and unconscious information processing. Consequently, the bottom-up network is more affected. At the same time, goal-directed (top-down) attention is more intact. Therefore, high-level, conscious, and voluntary mental processes may act as a compensatory reserve in rehabilitation work [14].

In patients with left neglect, visual search and information processing predominantly occur on the right side of the visual field. At a certain stage during visual scanning, such patients experience the phenomenon of “search satisfaction”, so they stop exploring the visual space on the left side. However, when the patient’s attention is specifically drawn to the left half of the visual field, he or she often notices objects located there [15,16]. It is worth noting that, according to M. Eglin et al., the number of objects within a limited visual field affects the degree of neglect syndrome manifestation, but the size of the display area alone does not have a significant effect [17].

In the diagnostics of neglect, visual search tasks are incorporated into various instruments, mainly based on the type of correction tests (stimuli only or stimuli with distractors). At the same time, there are virtually no methods that take into account the different levels of object load in the right and left 111 halves of the visual field. However, the degree to which the visual field is loaded with the number of objects can have a serious impact on the efficiency of visual search. This issue is relevant because the sensitivity of the techniques and tests used to assess visual neglect can vary greatly for the same patient. It is logical to assume that visual search depends on a number of different factors and conditions under which visual search is performed. One of these factors is apparently the object load in the visual field, which reflects the conditions of a person’s real-life activity. To test this assumption, a special technique was developed to account for the degree of object load in the right and left hemifields.

The present study examines the bottom-up network, with a particular focus on the impact of the number of stimuli in the right hemifield on the same visual search for target stimuli. The aim of the present study was to examine visual search performance in the left (neglected) side of space as a function of the number of stimuli present in the right hemifield. It is assumed that the efficiency of visual search in the left (ignored) visual hemifield in patients with neglect syndrome depends on the degree of object load in the right visual hemifield. Based on this assumption, an experiment was conducted to investigate the impact of the quantity of material in the right half of the space on the ability to locate items on the left.

It is proposed that patients with neglect syndrome, when searching for stimuli in a sequential manner from right to left, will demonstrate a depletion of attention resources, resulting in the avoidance of stimuli in the left hemifield. Thus, the hypothesis of this study is that an increase in the number of objects presented in the right visual hemifield will lead to a decrease in the number of objects found by patients in the left visual hemifield.

## 2. Materials and Methods

The present study was conducted at the Department of Medical Rehabilitation for Patients with Impaired Central Nervous System Function at the Pirogov National Medical and Surgical Center of the Ministry of Health of the Russian Federation. All patients signed a voluntary informed consent to participate in this study. This study was approved by the ethics committee of the Federal State Scientific Institution “Federal Scientific Center of Psychological and Multidisciplinary Research” (approval No. 2024/4).

A total of 50 individuals participated in this study. Inclusion criteria: lesion in the right hemisphere of the brain, clear state of consciousness, ability to remain in a sitting position and perform active tasks with at least one hand, absence of gross neurodynamic and regulatory disorders, normal or corrected vision, right-handedness, and no history of left-handedness and ambidexterity in the family. Exclusion criteria: speech impairment, severe dysregulation, dementia, visual impairment, and the presence of marked visual agnosia (e.g., object, color agnosia, prosopagnosia, or simultaneous agnosia).

This study included target and control groups. The target group included 30 patients with neurological conditions and left-sided neglect syndrome, aged between 36 and 75 years (58 ± 9.6). All patients underwent a neurological examination and were diagnosed with right hemispheric cerebral catastrophe: 28 stroke patients and 2 hemorrhage patients. The control group included 20 patients with neurological conditions without neglect syndrome, aged between 29 and 66 years (50.5 ± 10.7). These patients were also diagnosed with right hemispheric cerebral catastrophe: 19 stroke patients and 1 patient with hemorrhage. In both groups, a minimum of six months had elapsed between the onset of the neurological deficit and admission to rehabilitation.

All patients from both groups underwent a comprehensive neuropsychological examination using the A.R. Luria Schema, consisting of a number of tests, to assess the state of cognitive functions in patients after brain damage and to identify left-sided neglect. The examination was conducted between the second and fourth day of hospitalization, prior to the start of rehabilitation. Visuospatial difficulties were observed in the majority of patients.

**Figure 1:**
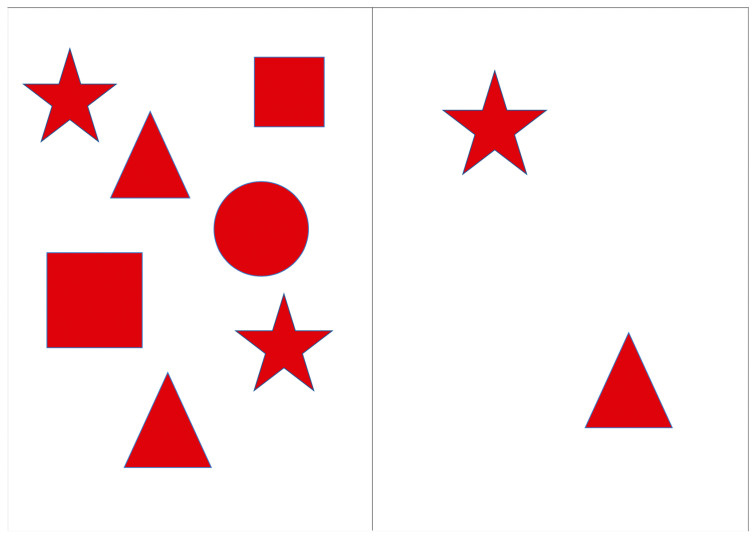
Series 1 of the Red Shapes test.

**Figure 2:**
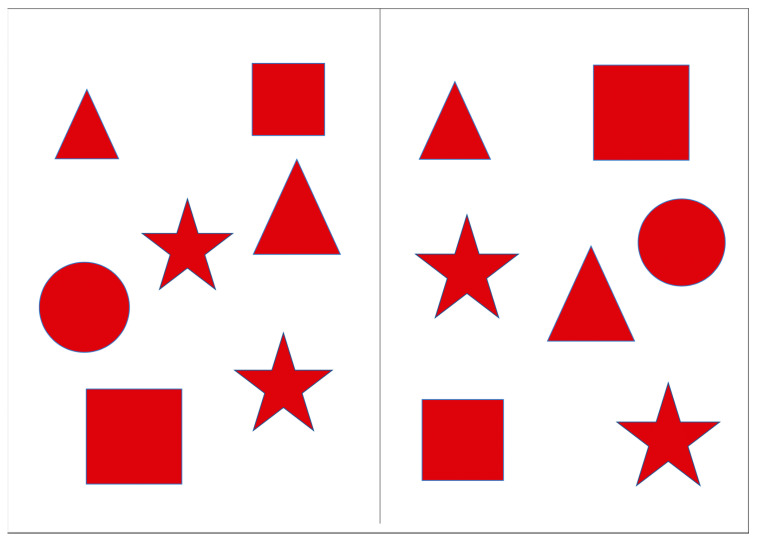
Series 2 of the Red Shapes test.

**Figure 3:**
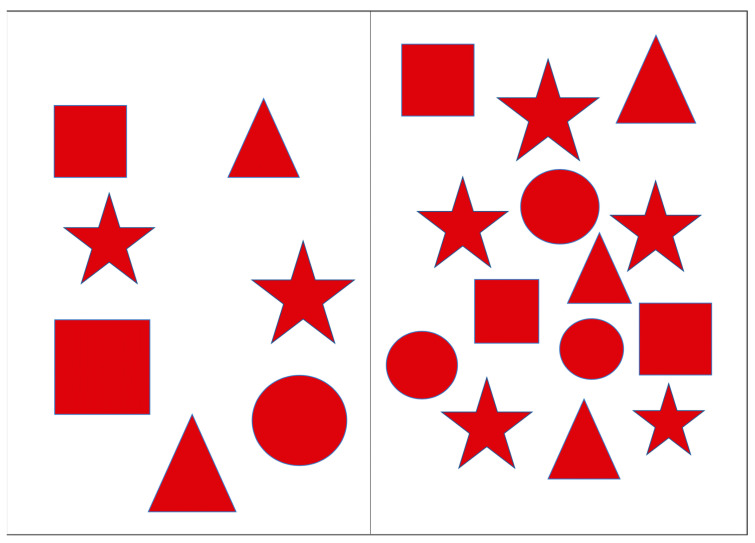
Series 3 of the Red Shapes test.

The Red Shapes test (RST) was designed to assess the efficiency of visual search in the left (ignored) visual hemifield when the load in the right hemifield was varied by changing the number of stimuli presented. The stimuli were presented on a portable screen with a 9.7″ diagonal, divided into two halves. Geometric figures (square, circle, triangle, and star) in red color were located on both halves of the screen. In order to prevent inadvertent activation of buttons that are not pertinent to the test procedure, a case was placed over the tablet in order to restrict access to these buttons. The patients were instructed to cross out all the figures they noticed as quickly as possible. During the presentation of the instructions, the experimenter demonstrated the method of crossing out the lines by drawing a narrow strip with their finger on a blank screen. Three successive series were created, each differing in the number of figures presented on the right half of the screen; the number of figures on the left side was constant, with only their locations changing. Series 1: the number of elements on the right (2 pcs.) was smaller than on the left (7 pcs.) (Figure 1). Series 2: the number of elements on the right and left was the same (7 pcs.) (Figure 2). Series 3: the number of elements on the right (14 pcs.) was bigger than the number of elements on the left (7 pieces) (Figure 3). The total execution time of the technique and the number of shapes found on the left and right were estimated.

The scoring system of the technique corresponds to the number of figures found in each series. There were no time restrictions for completing the test.

The technique has been shown to be effective in assessing visuospatial search abilities in patients with neglect syndrome [18]. Among all the series of the RST, the third series, with 14 elements in the right hemifield and 7 in the left, is the most sensitive for detecting impairments in visuospatial search.

## 3. Results

**Figure 4:**
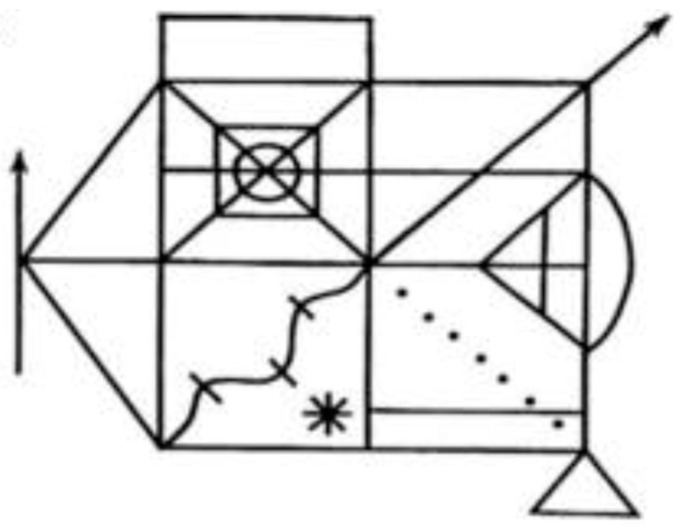
Taylor Complex Figure technique [19].

**Figure 5:**
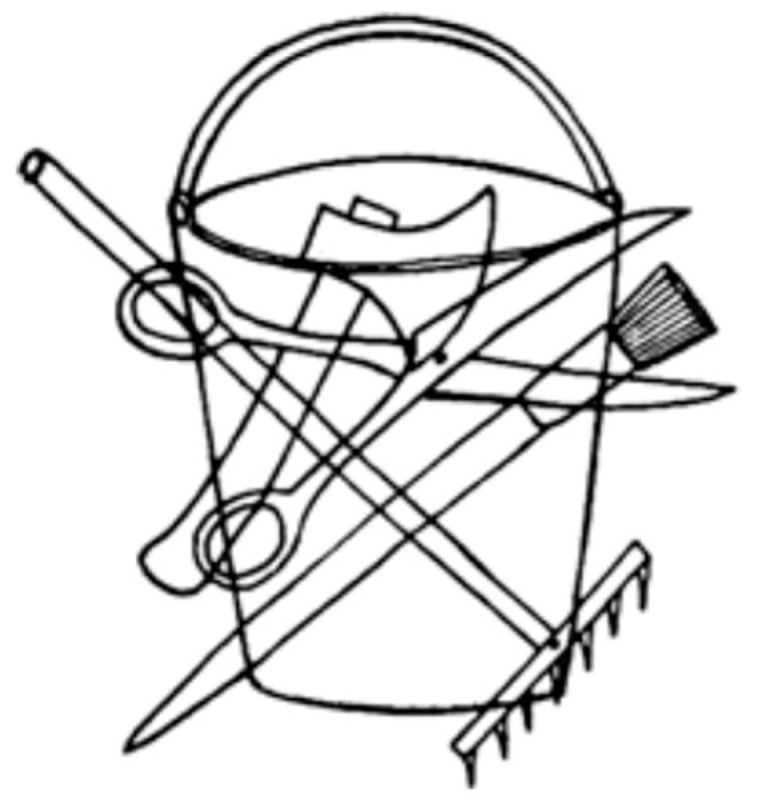
Poppelreuter–Ghent’s Overlapping Figures test [20].

Prior to the administration of the test, patients underwent a neuropsychological examination that also included an assessment of patients’ visual-spatial function. The objective of the tests employed to pre-diagnose patients with neglect syndrome was to assess visuospatial function, including neglect. This was conducted to ascertain whether patients exhibited gross visual processing impairments, including an object or simultaneous agnosia. The Taylor Complex Figure (Figure 4) has been demonstrated to be an effective tool for diagnosing visual neglect syndrome as well as other visuospatial impairments [19]. The Poppelreuter–Ghent’s Overlapping Figures test (Figure 5), among other things, allows for the evaluation of the integrity of perception in patients who have sustained brain damage [20].

A comparison of the experimental and control groups using the non-parametric Mann–Whitney criterion revealed significant differences on the majority of the test scales (e.g., Taylor figure, table and cube drawing, etc.).

The results from a number of diagnostic techniques indicated that the subjects in the control group exhibited higher indicators. It was initially hypothesized that the results of the target group patients would be significantly lower due to the presence of neglect syndrome, as the tests used were designed to diagnose this phenomenon.

The results of the RST demonstrated that the control group exhibited higher indicator values than the target group. This can be explained by the fact that the subjects of the target group ignored the left half of the screen, which led to the observed low indicator values.

Significant correlations were found between the indicators of the RST and other methods for assessing visuospatial functions.

RST, series 1. A moderate positive correlation (r = 0.434, *p* < 0.05) was observed between the number of elements identified on the left side when executing the RST and success in performing Poppelreuter–Ghent’s Overlapping Figures test. Similarly, a moderate negative correlation (r = −0.563, p ≤ 0.01) was found between this parameter and impaired integrity of perception of the object images. Additionally, moderate negative correlations were observed with the performance of the Taylor Complex Figure, particularly with regard to the following parameters: coordinate (r = −0.461, *p* ≤ 0.05), structural-topological (r = −0.387, *p* ≤ 0.05), and metric errors when performing this technique (r = −0.478, *p* ≤ 0.01). The presence of inverse correlations can be attributed to the fact that, in the evaluation system of the RST, the found stimuli were taken into account rather than omissions, while the parameters of performance in various techniques were evaluated on a three-point scale, where 0 indicates the absence of impairment, and 2 indicates its gross manifestation.

RST, series 2. Moderate positive correlations were found between the number of elements identified on the left side using the RST and performance on tasks such as

- The performance of the Taylor Complex Figure (r = 0.554, *p* < 0.01). Moderate negative correlations were also identified for performance parameters on this task, including metric errors (r = −0.482, *p* ≤ 0.01), coordinate errors (r = −0.44, *p* ≤ 0.05), and errors indicative of left-side neglect (r = −0.459, *p* ≤ 0.05).
- Poppelreuter–Ghent’s Overlapping Figures test (r = 0.412, *p* < 0.05). A moderate negative correlation was found in relation to the parameter of fragmentary perception of object images (r = −0.402, *p* ≤ 0.05).

RST, series 3. Moderate positive correlations were observed between the number of items identified on the right side when executing the RST and the successful performance of techniques such as

- The performance of the Taylor Complex Figure (r = 0.397, *p* < 0.05). Moderate negative correlations were obtained for parameters such as left-sided neglect (r = −0.368, *p* ≤ 0.05), metric errors (r = −0.374, *p* ≤ 0.05), and structural-topological errors (r = −0.418, *p* ≤ 0.05).
- Independent cube drawing (r = 0.411, *p* < 0.05).
- A moderate negative correlation was observed between the impaired integrity of perception of object images when performing Poppelreuter–Ghent’s Overlapping Figures test (r = −0.41, *p* ≤ 0.05) and the number of items found on the right side of the RST.

Additionally, moderate positive correlations were obtained between the number of elements found on the left side of the RST and the success in performing tests such as

- Copying of the Taylor Complex Figure (r = 0.384, *p* ≤ 0.05). Moderate negative correlations were obtained for the following performance parameters of this technique: metric errors (r = −0.382, *p* ≤ 0.05) and left-sided neglect (r = −0.507, *p* ≤ 0.05).
- Poppelreuter–Ghent’s Overlapping Figures test (r = 0.457, *p* ≤ 0.05). A moderate negative correlation was obtained for the parameter of impaired integrity of perception of object images (r = −0.463, *p* ≤ 0.05).

The analysis of the results demonstrated the absence of statistically significant correlations in the control group for both the specialized tests and the RST.

The data obtained may indicate that in the target group, neglect is accompanied by visual-spatial disorders such as a lack of perceptual integrity, alterations in the structure of space, and changes in metric and coordinate representations.

This paper focuses on the analysis of the results obtained from the execution of the RST.

Statistical analysis of the RST data was conducted using the IBM SPSS Statistics 23.0 system and included the Shapiro–Wilk’s criterion and the non-parametric Friedman statistical test.

The Shapiro–Wilk test was used to determine the normal distribution of outcome measures [21,22]. The results of the verification showed that the data for all measures, except for the number of found stimuli on the right (*p* = 0.056) and time (*p* = 0.18) in Series 3, did not follow the laws of a normal distribution. In this regard, the non-parametric Friedman’s criterion was used [23]. The Friedman Test was used to evaluate whether there were changes in RST performance as a function of the test series.

As part of the statistical analysis, we noticed that when calculating the significance of the differences in the “number of elements found on the right”, the number of elements varied across the series. Therefore, it was decided to convert the points obtained into fractional indicators as follows: the number of elements found on the right divided by the total number of figures on the right within the series presented. Similar mathematical manipulations were carried out for the “number of elements found on the left”, although the total number of figures presented did not change from series to series. The results of the statistical analysis for the target group are shown in Table 1.

Table 1 illustrates that all three measures (proportion of stimuli located on the right, proportion of stimuli located on the left, and series completion time) demonstrate significant changes between series in the group of patients diagnosed with neglect syndrome. Kendall’s W indicates large effects (W > 0.5) for the Friedman test across all indicators.

In contrast to the target group, no significant differences in performance between series were observed in the control group, with the exception of the time taken to complete the series (see Table 2). 

Differences in completion time can be attributed to the increase in the number of stimuli required to be crossed out.

**Figure 6:**
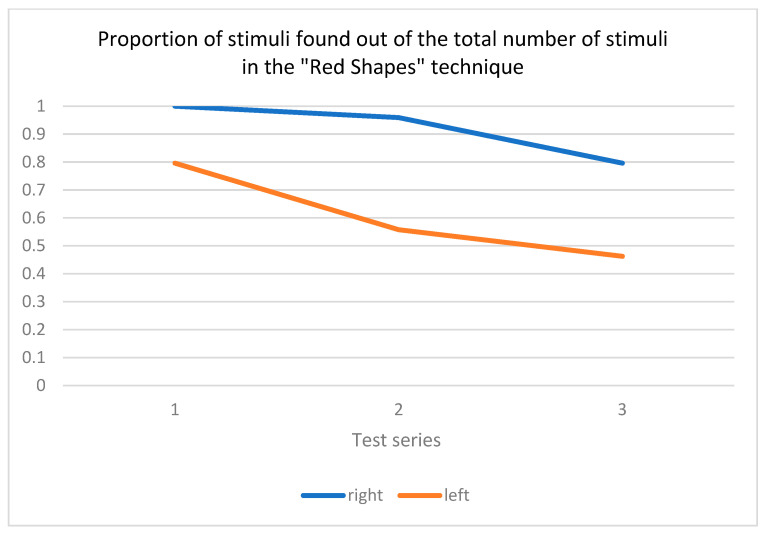
Proportions of stimuli found from the total number of stimuli in the Red Shapes test (by parameters such as “left” and “right”); the *X*-axis represents the study series, and the *Y*-axis represents the proportion of stimuli found on the right or left relative to the total number of stimuli on the right or left, respectively.

Figure 6 shows how the success rates of the RST change over time. For this visualization, the same scores were used as those employed to calculate the Friedman criterion (the proportion of stimuli found ono the right or left relative to the total number of stimuli to the right or left), as each series has a different number of figures to the “right”.

**Figure 7:**
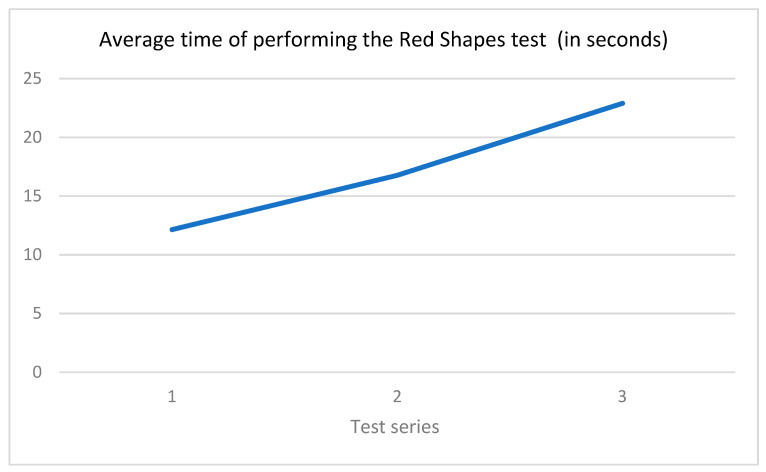
Average execution time for the Red Shapes test; the *X*-axis represents the study series, and the *Y*-axis represents the time (in seconds) spent performing the test.

Figure 7 shows the change in the time taken to complete the RST across the series.

Figure 7 shows that from series to series, there is an increase in the time taken to find the shapes. This is apparently due to an increase in the number of stimuli presented and, consequently, an increase in the visual load, while the attention span is limited.

## 4. Discussion

As a result of the analysis, significant differences (*p* < 0.001) were found in the visual search results for all indicators measured within the framework of the RST, namely the proportions of stimuli found to the right/left relative to the total number of elements on the right/left, as well as the time spent on each series.

Figure 4 shows that the number of stimuli found in both the right and left visual hemifields decreased from series to series. During the implementation of the RST test, participants sometimes ignored not only the left visual hemifield but also the central part of the visual field, which affected the success of the search for figures located in the right hemifield. This was particularly evident in the third series (fourteen elements on the right and seven elements on the left). This means that the success of finding stimuli in the left half of the visual field deteriorates as a function of the number of elements in the right half, i.e., the more shapes in the right half of the visual field, the fewer shapes the patient finds on the left.

An interesting result was the decrease in the effectiveness of the visual search for figures on the right. This is probably due to the fact that some of the objects in the center of the visual field are ignored. These data require an explanation and additional experiments. It is possible that the decrease in the number of stimuli found on the right is related to the direction of the patient’s gaze [24,25]. In this case, the location of the left and right hemifields becomes relative and depends on the point of fixation of the gaze. Therefore, the central part of the screen turns out to be vulnerable to negation, as it falls into the left and right hemifields. As the number of stimuli on the right side of the visual field increases, the attention load also increases. By focusing on the right side, patients begin to ignore the left side, including the central area, when the entire visual field is perceived. This finding may also be attributed to visual extinction, which exhibits comparable mechanisms to neglect but is manifested at the stimulus level and frequently co-occurs with neglect [26]. Patients may have encountered difficulties in detecting contralesional stimuli when presented with ipsilesional stimuli within the ipsilesional visual hemifield, resulting in the omission of some figures.

Furthermore, it is important to acknowledge that the efficacy of neglect patients in detecting stimuli in the left hemifield may be influenced by patient attitudes that are presumably different in each series, reflecting goal-directed (top-down) processes. In particular, when there is a minimal number of stimuli in the right hemifield, the patient may assume that these are not the only stimuli in the task, thereby exploring the space with increased activity and demonstrating greater attention to the left hemifield.

### Limitations

(1) Small sample size and low achievability of the target group

Neglect syndrome is a rare neuropsychological disorder observed in patients with lesions in the right cerebral hemisphere. In order to increase the size of the experimental group during this study, new patients fulfilling the inclusion criteria had to wait for a considerable amount of time.

(2) Attempts to use non-clickable interface elements

During the test execution on the electronic tablet, patients performed the target actions as well as accidentally pressing unnecessary buttons, such as the lock, switch off, and volume change buttons. In this case, the psychologist had to use the case and teach patients how to use the tablet.

(3) Differences in the manifestation of neglect

The presentation of neglect, including visual-spatial neglect, may vary depending on the circumstances and the distance involved. This article focuses on visuospatial neglect observed at short distances and assessed using complex neuropsychological evaluations and paper-and-pencil tests. Further studies are required to ascertain whether this phenomenon also manifests in other forms of neglect, including right-sided neglect.

(4) Methods used to collect the data

Further comparison of the results obtained with other methods of researching left-sided neglect would be beneficial, particularly an evaluation of shifts in attention focus and subjective changes in the structure of space in patients with neglect syndrome.

(5) Complexity of neglect syndrome

Neglect syndrome is a serious condition that frequently co-occurs with other neuropsychological impairments, particularly visual-spatial deficits. Further studies are required to investigate the performance of individuals with neglect compared to those with other distinct neuropsychological conditions.

## 5. Conclusions

In conclusion, the results obtained in this study can be explained by the phenomenon of SSM (subsequent search misses) [11], in particular by the theory of attentional resource depletion during visual search tasks [27,28]. The concept of resource depletion is supported by the increased influence of the “visual noise” factor on the success of detecting the second target stimulus after finding the first one. This phenomenon manifested itself in the present study in the following way: as the number of figures presented on the right side increased, the number of figures found in the left half of the visual field decreased. In addition, our findings support the view of M. Eglin et al. that the number of objects within the restricted field of view has an influence on the extent to which neglect syndrome is manifested [17]. It is believed that the load in the right hemifield influences the degree of neglect in the left hemifield, acting as a distracting factor. Therefore, when developing rehabilitation measures aimed at overcoming neglect syndrome, it is necessary to pay attention to the right side of space. In particular, when organizing the patient’s space, it is important to ensure that the right side of the space (e.g., in a room) is not overloaded, as this will not encourage the patient to explore the left side of the space. Furthermore, when diagnosing and rehabilitating a patient with neglect, it is essential to consider the sensitizing effect of visual and attention load. The varying occupancy of the field or hemifield can be employed as a means of addressing varying levels of severity in neglect and neurodynamic features.

This study confirmed the hypothesis about the influence of the number of elements in the right visual hemifield on the search for elements in the left hemifield and raised a number of new questions related to the study of visual search characteristics as a function of different conditions, including visual field load and structure.

## Figures and Tables

**Table 1:** Results of statistical analysis using the Friedman test for the measures investigated in the Red Shapes test performance by the target group (proportions of found stimuli on the right/left relative to the total number of elements on the right/left, as well as the time spent performing the series).

	Right	Left	Time
N	30	30	30
Kendall’s W	0.76	0.52	0.68
χ2	45.40	31.29	40.49
df	2	2	2
Asymp. Sig.	0.000	0.000	0.000

**Table 2:** Results of the statistical analysis using the Friedman test for the measures investigated in the Red Shapes test performance by the control group (proportions of stimuli found on the right/left of the total number of elements on the right/left, as well as the time spent performing the series).

	Right	Left	Time
N	20	20	20
Kendall’s W	0.21	0.10	0.43
χ2	8.22	4.00	17.38
df	2	2	2
Asymp. Sig.	0.016	0.135	0.000

## Data Availability

The data presented in this study are available on request from the corresponding author.
